# A Hidden “Promoter”: *Schistosoma japonicum* soluble egg antigen activates MAPK/PI3K-AKT pathways and inhibits autophagy to facilitate colorectal cancer

**DOI:** 10.1128/iai.00696-25

**Published:** 2026-03-02

**Authors:** Jinyuan Zhang, Wenwen Zheng, Yiyan Gong, Xiaofang Jia, Hongli Liu, Fei Guan, Jiahui Lei

**Affiliations:** 1 Department of Pathogen Biology, School of Basic Medicine, Tongji Medical College and State Key Laboratory for Diagnosis and Treatment of Severe Zoonotic Infectious Diseases, Huazhong University of Science and Technology 12443, Wuhan, China; 2 Cancer Center, Union Hospital, Tongji Medical College, Huazhong University of Science and Technology 162789, Wuhan, China; 3 Union Hospital, Tongji Medical College, Institute of Radiation Oncology, Huazhong University of Science and Technology 36630, Wuhan, China; Tulane University, New Orleans, Louisiana, USA

**Keywords:** *Schistosoma japonicum*, soluble egg antigen, colorectal cancer, MAPK, PI3K-AKT, autophagy

## Abstract

Schistosomiasis affects approximately 250 million people globally. Eggs deposited in the colon are the main culprits causing pathological damage, with soluble egg antigen (SEA) being the most significant pathogenic factor in schistosomiasis. Colorectal cancer (CRC) is the most common type of gastrointestinal carcinoma. The colorectum is a common organ affected by both CRC and schistosomiasis. Clinical reports have demonstrated a correlation between the onset and poor prognosis of CRC and *Schistosoma japonicum* infection. However, there is still a lack of experimental evidence regarding the effect of schistosomiasis japonica on CRC. In this study, we found that *S. japonicum* SEA promoted the proliferation of CRC cells in a dose-dependent manner and upregulated the expression of Ki67. Moreover, SEA enhanced the migration and epithelial-mesenchymal transition of CRC cells. RNA sequencing analysis revealed that SEA activated the MAPK and PI3K-AKT signaling pathways while inhibiting autophagy in HCT116 cells, findings that were subsequently validated by Western blotting. In the murine xenograft model, SEA promoted tumor growth, increased the expression of Ki67 and Vimentin, and activated the MAPK and PI3K-AKT signaling pathways in tumor tissues. Overall, our findings demonstrate that *S. japonicum* SEA accelerates the malignant development of CRC both *in vitro* and *in vivo*, which is linked to the suppression of autophagy and the activation of MAPK and PI3K-AKT signaling. This is the first demonstration that the *S. japonicum* SEA participates in the induction of CRC-related signaling pathways, thus providing experimental evidence for the impact of schistosomiasis in endemic areas on CRC.

**IMPORTANCE:**

This research provides crucial experimental evidence bridging clinical observations and molecular mechanisms in schistosomiasis-associated colorectal cancer (CRC). For the first time, we demonstrate that *S. japonicum* soluble egg antigen (SEA) accelerates CRC malignancy both *in vitro* and *in vivo*. Mechanistically, SEA activates the key oncogenic MAPK and PI3K-AKT signaling pathways while inhibiting autophagy. These findings establish a functional link between a specific parasitic factor and host cancer-driving pathways. This work strongly suggests that chronic exposure to SEA in endemic areas constitutes a defined biological risk factor for CRC. Ultimately, this study provides experimental evidence for elucidating the impact of *S. japonicum* infection on CRC and further deepens our understanding of the role of SEA in promoting tumor progression. Our study highlights the importance of schistosomiasis control as a potential strategy for CRC prevention in endemic regions.

## INTRODUCTION

Schistosomiasis (bilharzia), a tropical disease caused by parasites of the genus *Schistosoma*, affects over 250 million people worldwide (1). *Schistosoma japonicum* is a major causative agent of human schistosomiasis. Following infection, female adults deposit eggs in the mesenteric vein. The development of egg granuloma and fibrosis as a result of the retention of countless eggs in the colonic wall is the hallmark pathological damage of schistosomiasis (2–4). The most significant pathogenic factor driving intestinal granulomas and fibrosis is the soluble egg antigen (SEA), which is released by eggs through micropores in the eggshell and enters surrounding tissues (2–6). This process leads to intestinal schistosomiasis, characterized by symptoms such as hematochezia, diarrhea, and abdominal pain (1, 2).

Infectious agents, including bacteria, fungi, viruses, and parasites, are estimated to account for approximately 15% of human malignancies (7). Notably, *Schistosoma haematobium* has been classified by the International Agency for Research on Cancer (IARC) as a Group 1 carcinogen due to its established role in the development of bladder cancer (8, 9). Clinical reports indicate that *S. japonicum* infection is associated with an increased incidence of colorectal cancer (CRC) and elevated mortality among CRC patients (10–13). However, there is currently a lack of experimental evidence regarding the effect of schistosomiasis japonica on CRC.

CRC is the most prominent carcinoma within the gastrointestinal tract, ranking third in global cancer incidence and accounting for approximately 10% of cancer-related fatalities annually (14). Pathogen infections and dysregulated signaling pathways are triggers for CRC. For instance, *Fusobacterium nucleatum* and *Blastocystis hominis* have been implicated as predisposing factors for CRC (15, 16). Among key signaling cascades, the MAPK and PI3K/AKT/mTOR pathways, which are widely engaged in cell proliferation, invasion, and metabolism, have a significant influence on the pathogenesis of CRC. Activation of these carcinogenic pathways is known to accelerate CRC pathogenesis (17, 18). A common downstream effector of both PI3K-AKT and MAPK pathways is mammalian target of rapamycin (mTOR), which inhibits autophagy upon activation (19). Although the impact of autophagy on CRC is context-dependent (20), evidence suggests that autophagy induction can inhibit the proliferation and survival of CRC cells. For example, it has been reported that curcumin can suppress the proliferation of HCT116 cells by promoting autophagy (21). However, whether and how schistosome infection, particularly *S. japonicum* SEA, modulates these pathways to influence CRC remains unclear.

Based on the previous literature research, this work aimed to investigate the effects of SEA of *S. japonicum* on CRC through both *in vitro* and *in vivo* experiments, as well as the underlying mechanisms. The results would provide experimental evidence for clarifying the impact of schistosomiasis on the development and progression of CRC.

## MATERIALS AND METHODS

### Cell culture

The human and mouse CRC cell lines HCT116, SW480, MC38, and CT26 were obtained from the Cell Bank of the Chinese Academy of Sciences (Shanghai, China). DMEM medium (Servicebio, China) was used to cultivate the SW480, HCT116, and MC38 cell lines. RPMI-1640 medium (Servicebio, China) was used to cultivate the CT26 cell line. All cell lines were handled according to standard protocols and cultured in media supplemented with 10% fetal bovine serum (Servicebio, China). Cells were incubated at 37°C in a humidified environment with 5% CO_2_.

### Ethics statement, animals, and parasites

All animal experiments were performed in strict accordance with the Institutional Animal Care and Use Committee at Tongji Medical College, Huazhong University of Science and Technology. All infection procedures and animal sacrifices were performed under anesthesia.

Six-week-old male athymic nude mice were acquired from Liaoning Changsheng Biotechnology Company (Liaoning, China). All mice were kept in a standard specific pathogen-free research animal facility. *Oncomelania hupensis* infected with *S. japonicum* was provided by the Gong'an *Oncomelania hupensis* Ecological Station (Gong'an, China).

### Preparation of *S. japonicum* SEA

Forty-five days post-infection, rabbits that had been exposed to *S. japonicum* cercariae via abdominal skin inoculation were euthanized, and their livers were aseptically collected. Liver homogenates were filtered through a series of sieves with pore sizes of 425, 125, and 48 μm to isolate schistosome eggs. Eggs retained on the 48 μm sieve were rinsed with sterile normal saline, collected, and centrifuged for 5 min at 45 × *g*. After centrifugation, the eggs were resuspended in saline and homogenized using a glass homogenizer to prepare SEA, which was then aliquoted and kept at −80°C until use.

### Animal tumor xenograft model

Male athymic nude mice were given subcutaneous injections of HCT116 cells (5 × 10^6^ each mouse) into their right flanks. Starting on day 2 post-cell inoculation, mice in the treatment group were intraperitoneally injected with 50 μg of SEA dissolved in 100 μL of phosphate-buffered saline (PBS) at 4-day intervals, while those in the control group were administered an equal volume of PBS alone. The mice’s body weight was monitored throughout the experimental period. Tumor diameters were measured to track tumor development. After measuring the tumors’ width (*W*) and length (*L*), the tumor volume was calculated using the formula: volume = (*W* × *L*)^2^/2. On day 28 following the injection, all mice were sacrificed. Tumors were excised, weighed, and photographed immediately for subsequent analyses.

### Cell proliferation assay

HCT116, SW480, MC38, and CT26 cells were plated in 96-well plates at a density of 5 × 10^3^ cells per well for the proliferation assay. After a 12-h incubation, the cells were exposed to SEA at the predetermined concentrations. The CCK-8 assay was used to measure cell proliferation in accordance with the manufacturer’s instructions (Servicebio, China). The absorbance of the reaction mixture was measured at a wavelength of 450 nm using a microplate reader (Thermo Fisher Scientific, Waltham, MA, USA).

### Colony formation assay

In six-well plates, 500 CRC cells were seeded into each well. The cells were cultured in complete medium supplemented with 10 μg/mL SEA for a total duration of 12 days, with the medium refreshed every 4 days. Subsequently, the cells were then rinsed with PBS, fixed with 4% paraformaldehyde solution, and labeled with 0.1% crystal violet (Servicebio, China).

### Wound healing assay

HCT116 cells (2 × 10^5^ cells per well) were seeded in six-well plates for the wound healing assay. After 24 h of incubation, cells were starved in serum-free medium overnight. After scratching the cells in layers with a sterile 200 μL pipette tip, cells were rinsed with PBS and then cultured in a serum-free medium containing 10 μg/mL SEA. At 0, 12, 24, and 48 h after the scratches, images of the wound areas were captured to monitor the cell migratory process, and the length of the wounds was measured.

### Transwell assay

A 24-well cell culture insert (NEST, China) with an 8 μm pore size polyethylene terephthalate membrane was used to measure the cell movement. HCT116 cells (5 × 10^4^ per chamber) were cultured in serum-free medium with 10 μg/mL SEA and then placed into the upper chamber of the transwell inserts. The bottom chamber was filled with a medium containing 20% FBS. The cells were incubated at 37°C in a humidified atmosphere with 5% CO₂ for 48 h. The chambers were then preserved with methanol and stained with 0.1% crystal violet solution. Images of the cells affixed to the membrane’s lower surface were captured.

### RNA sequencing analysis

HCT116 cells were cultured in medium supplemented with 10% FBS in the presence or absence of 10 μg/mL SEA for 24 h. Total RNA of HCT116 cells was extracted using the TRIzol reagent (Invitrogen, USA). RNA sequencing (RNA-seq) was performed by BGI Genomics (Shenzhen, China). Differentially expressed genes (DEGs) were analyzed by Dr. Tom.

### Flow cytometry

HCT116 cells were cultured in complete medium containing 10% FBS and 10 μg/mL SEA at the indicated time points. Following incubation, cells were collected, washed with precooled PBS, and fixed using a fixation/permeabilization solution for 30 min on ice. Then the cells were stained with PerCP/Cyanine5.5 anti-mouse/human Ki-67 antibody (Cat#151221, Biolegend, USA) for 30 min. Stained cells were analyzed with an Agilent/Novocyte 2060R flow cytometer. FlowJo software (Version 9.0) was used for all data analysis.

### Protein extraction and western blotting

Cells were cultivated in 6 cm diameter dishes and treated with 10 μg/mL SEA at specified time points. The proteins were obtained using RIPA Lysis Buffer (Beyotime, China) following a PBS wash. After being separated by 8% SDS-PAGE gels, the proteins were transferred onto a polyvinylidene difluoride (PVDF) membrane. Following blocking the membranes with 5% skim milk, the membranes were incubated overnight (18 h) at 4°C with the following antibodies: E-Cadherin (A20798, ABclonal), N-Cadherin (A3045, ABclonal), Vimentin (A19607, ABclonal), α-SMA (A2235, ABclonal), Snail (A5243, ABclonal), Twist (A15596, ABclonal), AKT1 (A17909, ABclonal), Phospho-AKT1 (AP0980, ABclonal), PI3K (A22730, ABclonal), mTOR (A2445, ABclonal), Phospho-mTOR (AP0115, ABclonal), ERK1/2 (A4782, ABclonal), Phospho-ERK1/2 (AP0974, ABclonal), p38 (A5049, ABclonal), Phospho-p38 (AP1508, ABclonal), LC3B (A19665, ABclonal), p62 (A19700, ABclonal), ATG5 (A11427, ABclonal), ATG7 (A21895, ABclonal), Phospho-PI3K (#13857, CST), β-actin (AC038, ABclonal), and GAPDH (AC001, ABclonal). The membranes were incubated with horseradish peroxidase (HRP)-conjugated mouse anti-IgG (BL001A, Biosharp) or HRP-conjugated rabbit anti-IgG (BL003A, Biosharp) secondary antibodies prior to the development of the membranes by ECL reagent (Servicebio, China). Then, the obtained signals were subjected to semi-quantitative analysis using Fiji ImageJ Software.

### Immunohistochemistry

As previously described (22), immunohistochemistry staining was carried out. Briefly, tumor tissues excised from the mice were fixed in 4% paraformaldehyde solution and then embedded in paraffin. Anti-Ki67 and anti-Vimentin antibodies (Servicebio, Wuhan, China) were used to label the sections. Fiji ImageJ was used to quantify the positively stained cells.

### Statistical analysis

All data were statistically analyzed by GraphPad Prism 8.0 software and expressed as mean ± standard deviation. Comparisons between two groups were conducted using paired t-tests or unpaired t-tests, while comparisons among multiple groups were analyzed by one-way analysis of variance (ANOVA). The significant difference levels were indicated by ^*^P < 0.05, ^**^ *P* < 0.01, ^***^ *P* < 0.001, and ^****^P < 0.0001.

## RESULTS

### The proliferation of CRC cells is promoted by SEA *in vitro*

**Fig 1:**
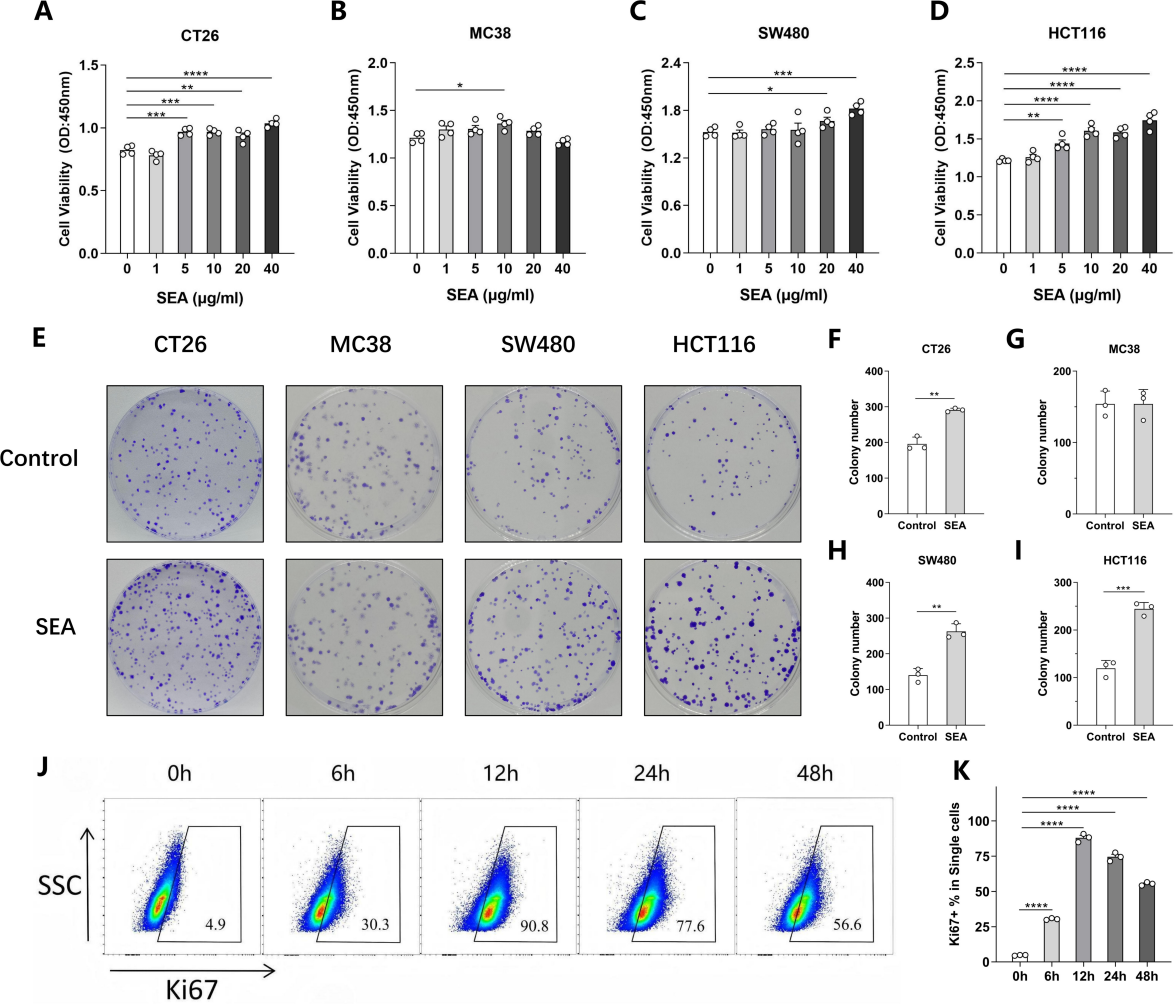
*S. japonicum* SEA promotes the proliferation of CRC cells *in vitro*. (**A–D**) The effect of different concentrations of SEA on the proliferation of CT26 (**A**), MC38 (**B**), SW480 (**C**), and HCT116 (**D**) was detected by a CCK-8 assay. (**E–I**) Representative colony formation images (**E**) and statistical graphs of CT26 (**F**), MC38 (**G**), SW480 (**H**), and HCT116 (**I**) stimulated with 10 μg/mL SEA. (**J and K**) Representative flow cytometry images (**J**) and statistical graph (**K**) of HCT116 cells stimulated with 10 μg/mL SEA at different time points. Each experiment was performed at least three times. The data are expressed as mean ± standard deviation. ^*^P < 0.05, ^**^P < 0.01, ^***^P < 0.001, and ^****^P < 0.0001.

To explore the impact of *S. japonicum* SEA on the proliferation of CRC cells, a CCK-8 assay was performed to investigate the proliferation activity of CRC cells. The results showed that SEA dose-dependently increased the viability of SW480, HCT116, and CT26 cells, respectively (Fig. 1A, C and D). Interestingly, at a concentration of 10 μg/mL SEA, the viability of MC38 cells was enhanced, whereas it was reduced at 40 μg/mL SEA (Fig. 1B). A colony formation assay was further used to verify the effect of SEA on CRC cell proliferation, and the results were consistent with those of the CCK-8 assay. Specifically, SEA stimulation promoted the formation of cell colonies in CT26, SW480, and HCT116 cells (Fig. 1E through I). Since the proliferation effect of HCT116 cells was the most significant after SEA stimulation, the cell line was selected as the experimental subject for subsequent experiments. Flow cytometric analysis of HCT116 cells revealed that the proportion of Ki67^+^ cells in HCT116 cells was increased and reached the peak at 12 h after SEA stimulation (Fig. 1J and K). Collectively, these findings demonstrate that *S. japonicum* SEA promotes CRC cell proliferation *in vitro*.

### The migration and epithelial-mesenchymal transition of CRC cells are promoted by SEA

**Fig 2:**
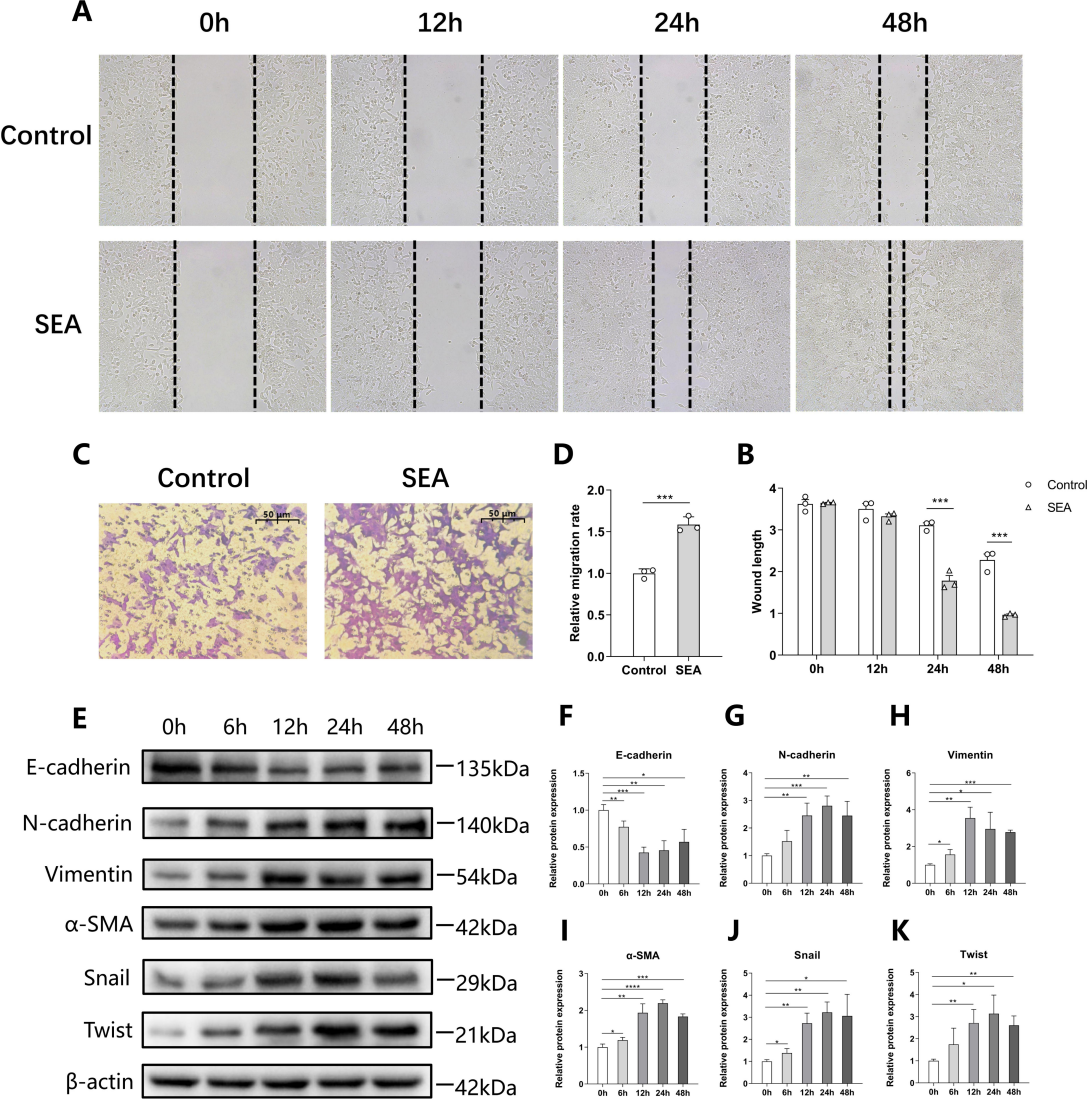
*S. japonicum* SEA promotes the migration and EMT of CRC cells *in vitro*. (**A and B**) Representative wound healing images (**A**) and statistical graph (**B**) of HCT116 cells stimulated with 10 μg/mL SEA at different time points. (**C and D**) Representative Transwell images (**C**) and statistical graph (**D**) of HCT116 cells stimulated with 10 μg/mL SEA. (**E–K**) Representative Western blotting images (**E**) and statistical graphs (**F–K**) of EMT markers of HCT116 cells stimulated with 10 μg/mL SEA at different time points. Each experiment was performed at least three times. The data are expressed as mean ± standard deviation. ^*^P < 0.05, ^**^P < 0.01, ^***^P < 0.001, and ^****^P < 0.0001.

As cell migration is an important characteristic reflecting CRC cell malignancy, wound healing and Transwell assays were carried out to assess the effect of SEA on HCT116 cell migration. The results of the wound healing assay showed that SEA promoted the wound healing of HCT116 cells (Fig. 2A and B), and the Transwell assay further confirmed that SEA accelerated the migration capacity of HCT116 cells (Fig. 2C and D). Epithelial-mesenchymal transition (EMT) plays a significant role in tumor development, especially in tumor metastasis. Therefore, we detected the expression of EMT-related markers by Western blotting. The results indicated that SEA upregulated the protein expression of mesenchymal markers (N-cadherin, Vimentin, α-SMA) and EMT transcription factors (Snail and Twist), while downregulating the expression of epithelial marker E-cadherin (Fig. 2E through K). These findings indicate that SEA promotes EMT in HCT116 cells.

### SEA activates the MAPK and PI3K-AKT pathways in CRC cells

**Fig 3:**
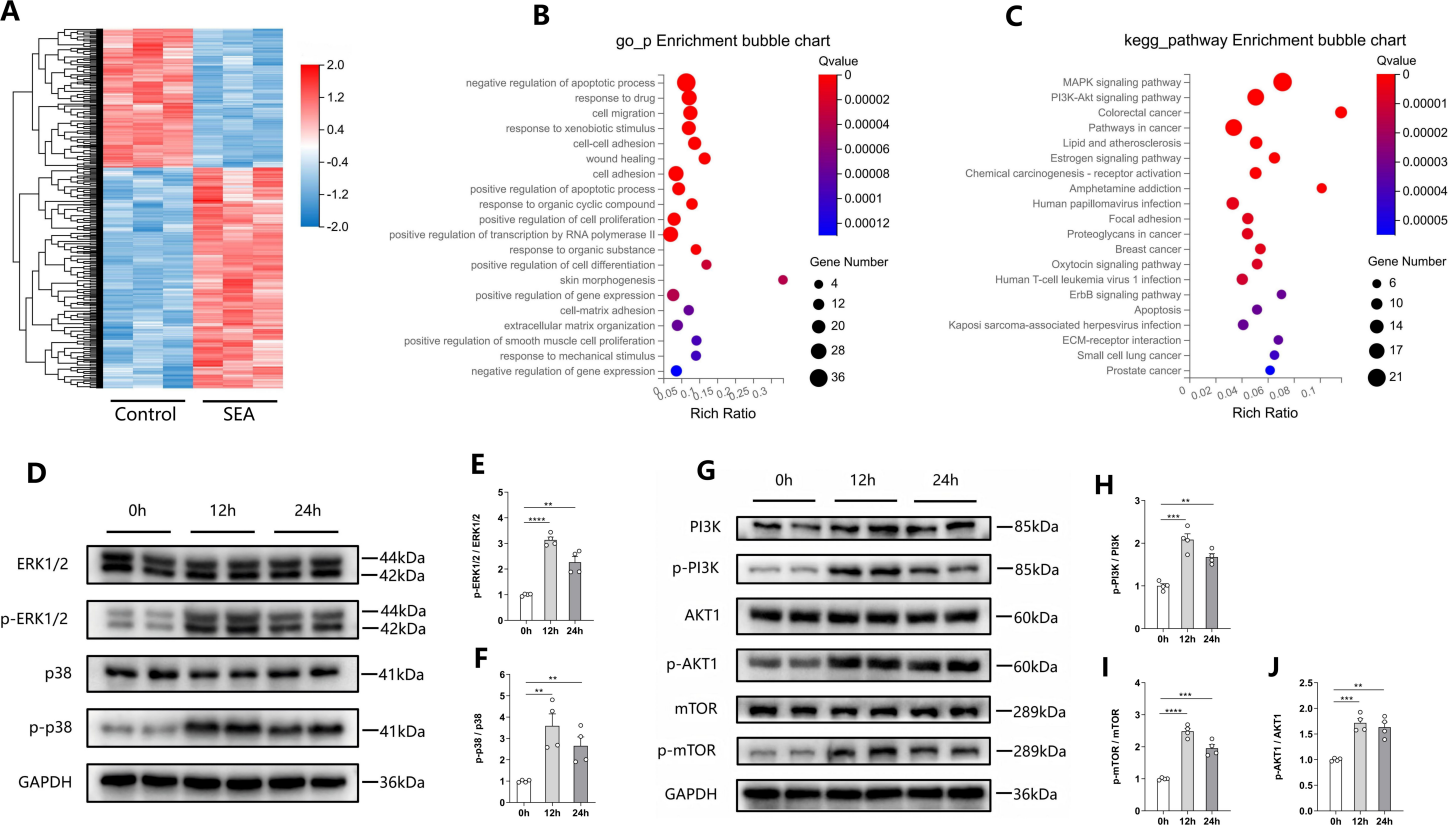
*S. japonicum* SEA activates the MAPK and PI3K-AKT pathways in CRC cells. (**A**) A heatmap of DEGs in 10 μg/mL SEA stimulated and control HCT116 cells. The log2 values were calculated for each sample by normalizing the data to the number of reads alone (*P* < 0.05). (**B**) GO biological processes enrichment bubble chart of DEGs. (**C**) KEGG pathway enrichment bubble chart of DEGs. (**D–F**) Representative Western blotting images (**D**) and statistical graphs (**E and F**) of the MAPK pathway marker of HCT116 cells stimulated with 10 μg/mL SEA at different time points. (**G–J**) Representative Western blotting images (**G**) and statistical graphs (**H–J**) of the PI3K-AKT pathway marker of HCT116 cells stimulated with 10 μg/mL SEA at different time points. Each experiment was performed at least three times. The data are expressed as mean ± standard deviation. ^**^P < 0.01, ^***^P < 0.001, and ^****^P < 0.0001.

Next, we conducted RNA-Seq analysis to investigate the molecular mechanism involved in the promotion of CRC by SEA *in vitro*. A heatmap was generated after analyzing DEGs (Fig. 3A). A total of 448 DEGs were identified between SEA-stimulated HCT116 cells and their control group, including 275 upregulated and 173 downregulated genes. To explore the potential biological functions of these DEGs, Gene Ontology (GO) biological process enrichment was conducted. The results showed that SEA affected multiple important biological processes, including negative regulation of the apoptotic process, negative regulation of apoptosis, cell migration, response to xenobiotic stimulus, cell-cell adhesion, wound healing, and positive regulation of cell proliferation (Fig. 3B). Consistent with our previous *in vitro* experiment results, all of these are closely related to cell proliferation and migration. Furthermore, Kyoto Encyclopedia of Genes and Genomes (KEGG) pathway enrichment analysis was performed to explore the molecular pathways through which SEA regulates CRC cells. We discovered that the enriched pathways were mainly concentrated in the PI3K-AKT and MAPK signaling pathways (Fig. 3C). Western blotting analysis was further performed to verify these findings, confirming that SEA stimulation activated the MAPK and PI3K-AKT signaling pathways in HCT116 cells, with the most significant activation observed at 12 h post-treatment (Fig. 3D through J). Taken together, the promoting effects of SEA on CRC cells are related to its activation of the MAPK and PI3K-AKT pathway.

### The promoting effect of SEA on the proliferation and migration of CRC cells is related to its inhibition of autophagy

**Fig 4:**
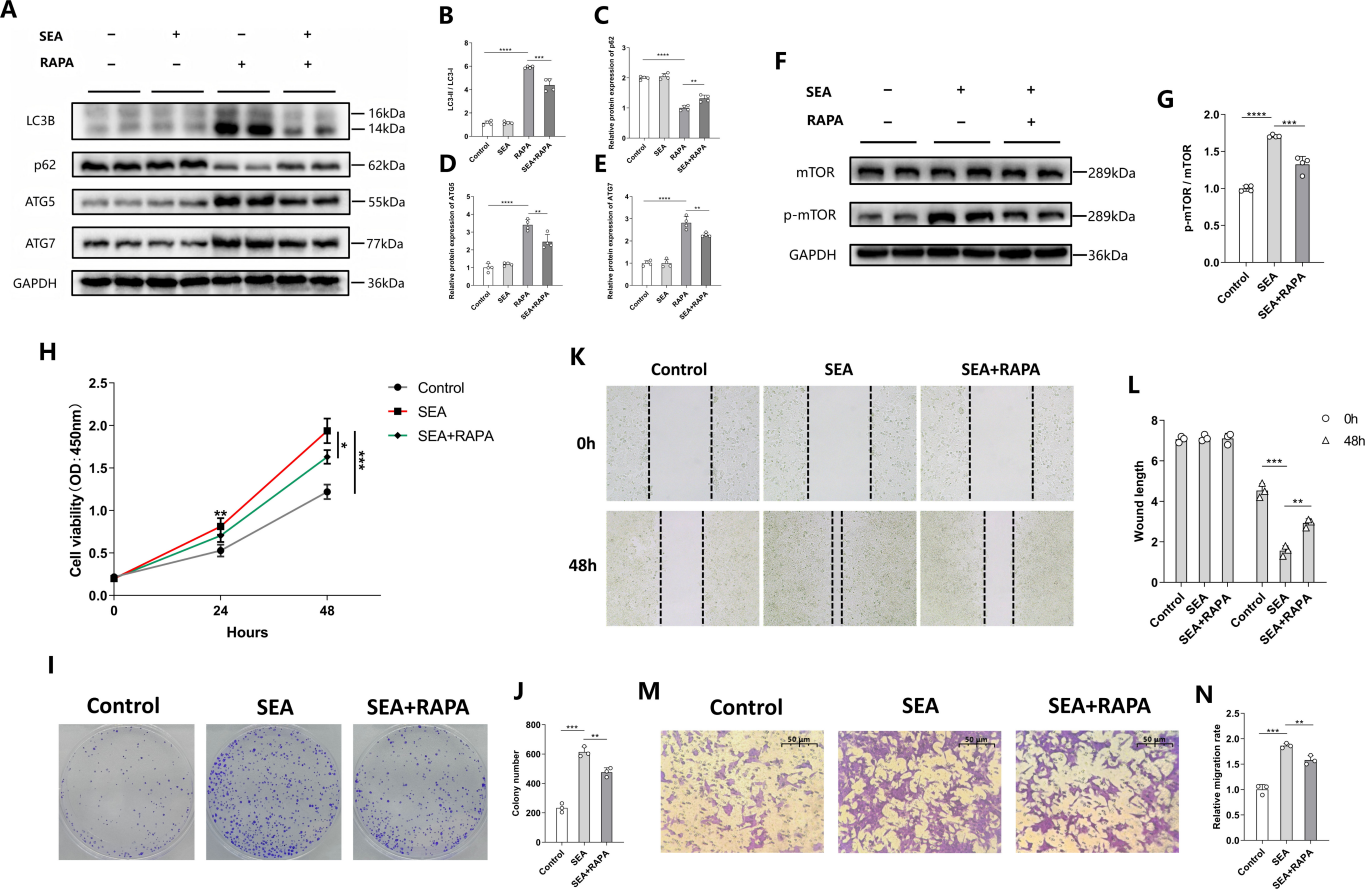
*S. japonicum* SEA promotes the proliferation and migration of CRC cells by inhibiting autophagy. (**A–E**) Representative Western blotting images (**A**) and statistical graphs (**B–E**) of the autophagy marker of HCT116 cells stimulated with 10 μg/mL SEA or 100 nM RAPA for 12 h. (**F and G**) Representative western blotting images (**F**) and statistical graph (**G**) of the activation of mTOR of HCT116 cells stimulated with 10 μg/mL SEA or 100 nM RAPA for 12 h. (**H**) CCK-8 assay to analyze the effect of 100 nM RAPA on the proliferation of HCT116 cells stimulated with 10 μg/mL SEA. (**I and J**) Representative colony formation images (**I**) and statistical graph (**J**) of HCT116 cells treated with 10 μg/mL SEA or 100 nM RAPA. (**K and L**) Representative wound healing images (**K**) and statistical graph (**L**) of HCT116 cells treated with 10 μg/mL SEA or 100 nM RAPA. (**M and N**) Representative Transwell images (**M**) and statistical graph (**N**) of HCT116 cells treated with 10 μg/mL SEA or 100 nM RAPA. Each experiment was performed at least three times. The data are expressed as mean ± standard deviation. ^*^P < 0.05, ^**^P < 0.01, ^***^P < 0.001, and ^****^P < 0.0001.

Since mTOR is a common downstream effector of the MAPK and PI3K-AKT pathways and its activation can inhibit autophagy (23), we further examined the impact of SEA on autophagy in HCT116 cells. The results indicated that SEA inhibited autophagy induced by rapamycin (RAPA) (Fig. 4A through E). To confirm whether the promotion of SEA on cell proliferation and migration was mediated by its regulation of autophagy, we examined the activation of mTOR in HCT116 cells treated with SEA in the presence or absence of the autophagy activator RAPA. The results showed that the activation of SEA on the mTOR signaling was partially reversed by RAPA (Fig. 4F and G). Consistent with this, CCK-8 and colony formation assay demonstrated that RAPA partially abrogated the stimulating effects of SEA on HCT116 cell proliferation (Fig. 4H through J). Additionally, the enhanced migration rate of HCT116 cells caused by SEA was also partially reversed by RAPA, as evidenced by Transwell and wound healing assays (Fig. 4K through N). Therefore, the promotion of SEA on CRC cells is associated with its suppression of autophagy.

### SEA promotes the growth of CRC in a murine xenograft model

**Fig 5:**
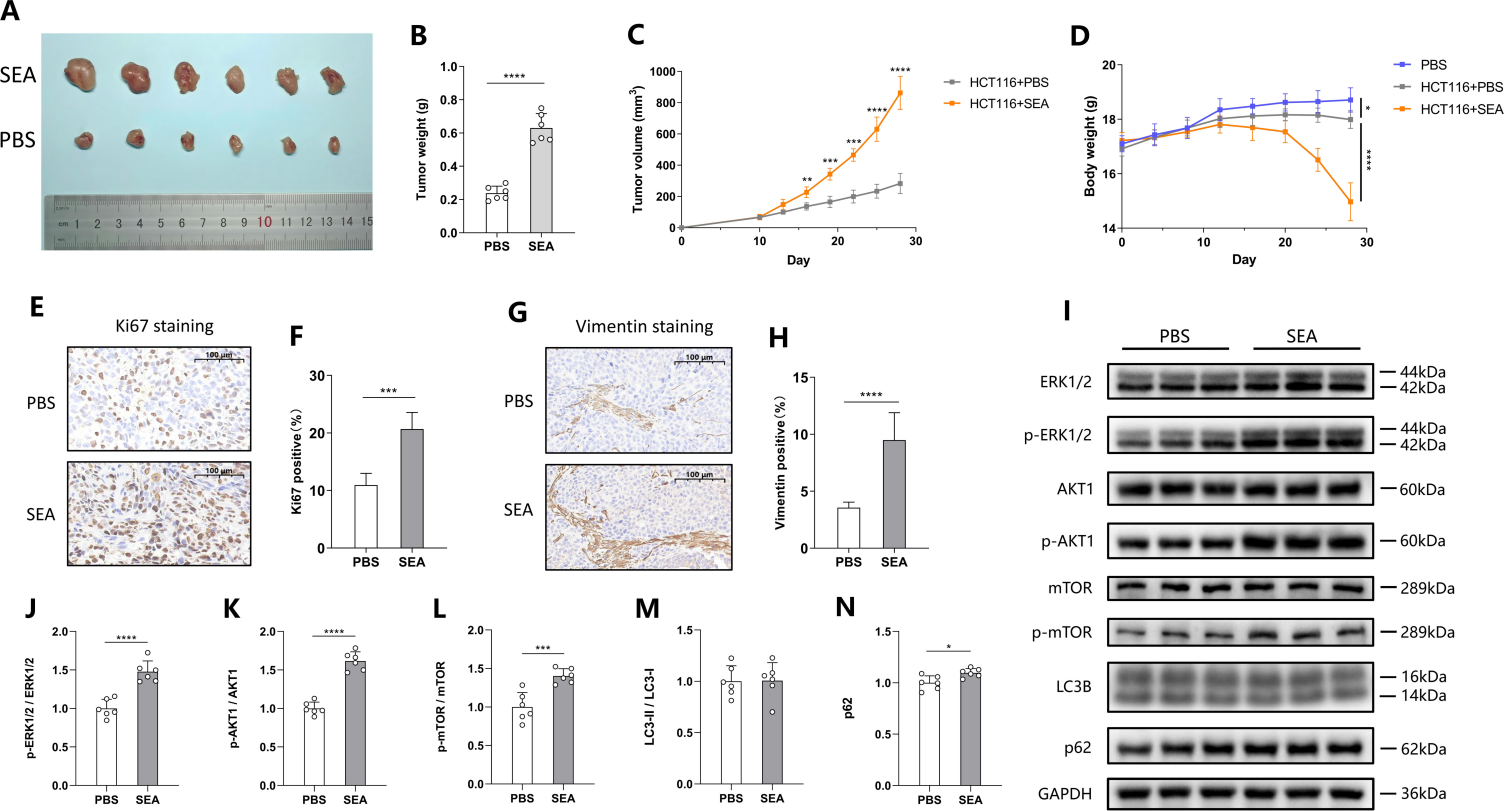
*S. japonicum* SEA exacerbates CRC in a murine xenograft model. (**A**) Image of the isolated mice tumors at day 28 after SEA treatment. (**B**) Tumor weight. (**C**) Tumor volume curve. (**D**) Body weight curve of mice. (**E and F**) Representative Ki67 staining images (**E**) and statistical graph (**F**) in tumor tissues. (**G and H**) Representative Vimentin staining images (**G**) and statistical graph (**H**) in tumor tissues. (**I–N**) Representative Western blotting images (**I**) and statistical graphs (**J–N**) of MAPK, PI3K/AKT/mTOR pathways, and autophagy markers in tumor tissues. Each experiment was performed at least three times. The data are expressed as mean ± standard deviation. ^*^P < 0.05, ^**^P < 0.01, ^***^P < 0.001, and ^****^P < 0.0001.

To evaluate the effect of SEA on CRC progression *in vivo*, we established a murine xenograft model by subcutaneously injecting HCT116 cells, followed by SEA treatment. The results indicated that SEA not only promoted tumor growth (Fig. 5A through C) but also reduced the murine body weight (Fig. 5D). Immunohistochemistry staining showed that SEA increased the expression of the proliferation marker Ki67 and the EMT marker Vimentin in tumor tissues (Fig. 5E through H). Next, we examined the activation of the MAPK and PI3K/AKT/mTOR signaling pathways in tumor tissues. In line with the *in vitro* findings, SEA activated the MAPK and PI3K/AKT/mTOR signaling pathways, while the effect of SEA on autophagy was not significant (Fig. 5I through N). These results confirm that SEA can promote the growth and EMT of CRC *in vivo* and activate the MAPK and PI3K/AKT/mTOR pathways, thereby facilitating the malignant progression of CRC.

## DISCUSSION

Parasitic infections are recognized as one of the risk factors for the development of malignant tumors in humans. Among them, *S. haematobium* is linked to bladder cancer, whereas *Opisthorchis viverrini* and *Clonorchis sinensis* are linked to cholangiocarcinoma (8, 9). New evidence suggests that *S. mansoni* is related to liver cancer and CRC (24–26). Clinical results indicate that CRC patients infected with *S. japonicum* have a shorter survival time (12, 13). However, the association between *S. japonicum* and CRC has thus far been limited to clinical reports. Here, we have confirmed that *S. japonicum* SEA can accelerate the malignant development of CRC both *in vitro* and *in vivo*.

SEA is the secretion production of miracidium in mature schistosoma eggs and represents the main active and pathogenic component of the parasite (2–6). *S. haematobium* SEA at a concentration of 6.25 μg/mL can stimulate the proliferation of urothelial cells and inhibit cell apoptosis (27). *S. mansoni* SEA induces endothelial cell proliferation in a concentration-dependent manner, while extracts of adult worms lack this effect (28). Additionally, *S. mansoni* SEA of 15 μg/mL significantly promotes the proliferation of the human hepatoma cell line HepG2 (26). To the best of our knowledge, we are the first to demonstrate that *S. japonicum* SEA can dose-dependently stimulate CRC cell growth.

Interestingly, when studying the impact of SEA on cell proliferation, the effect of SEA on MC38 cells is different from that on other cells, and there are two possible reasons for this. On the one hand, there is an intrinsic cell line heterogeneity. MC38 (a C57BL/6 mouse-derived carcinoma) has a unique genetic and molecular background. It is well documented that even oncogene-driven cancers exhibit differential dependency on key signaling pathways such as PI3K/AKT and MAPK/ERK, depending on the specific molecular context of each cell line (29). Inherent differences—such as receptor expression profiles, baseline pathway activity, or metabolic states—could fundamentally alter how each cell line responds to the complex stimuli of SEA. On the other hand, there is a dose-dependent biphasic signaling. The low-dose promotion and high-dose inhibition observed specifically in MC38 cells suggest a hormetic, biphasic response—a widely recognized safeguard against pathway overactivation in cell signaling systems (30). We hypothesize that low SEA doses preferentially activate pro-proliferative pathways in MC38 cells. At higher doses, the same or distinct SEA components may trigger countervailing signals (e.g., stress responses, feedback inhibition, or cyclin-dependent kinase inhibitor induction) that outweigh proliferative stimuli, resulting in growth suppression. This cell line-specific, dose-dependent complexity highlights the contextual nature of the effects of SEA.

Parasitic secretions can alter the host’s immune environment, trigger chronic inflammation, induce oxidative stress, cause gene mutations, and activate cellular signaling pathways (31), which are associated with carcinogenic mechanisms. For instance, granulin and glutathione S-transferase secreted by *O. viverrini* can activate the MAPK and AKT pathways, thereby promoting the proliferation of human cholangiocarcinoma cells (32, 33). Both of these proteins can strongly induce EMT (33, 34). *S. mansoni* SEA enhances oxidative stress in human liver cells and disrupts the cell cycle, and thus exerts a carcinogenic effect (25). In addition, *S. mansoni* SEA can upregulate the expression level of vascular endothelial growth factor (VEGF) in endothelial cells and promote angiogenesis (35, 36), which is a key process for tumor progression and metastasis (37). Another study reported that *S. mansoni* SEA promotes the expression of DNA damage repair markers and Cyclin D1 in human CRC cells, thereby promoting the progression of CRC (24). Additionally, *S. mansoni* SEA can induce the proto-oncogene c-Jun in human hepatoma cells and CRC cells (24, 25). The activation of c-Jun can promote cell proliferation and facilitate the transcription of the anti-apoptotic gene Bcl-2 (38). *S. mansoni* can activate the Wnt/β-catenin signaling in CRC cells, thereby promoting the progression of CRC (24). With respect to *S. japonicum*, the SEA component SjE16.7 acts as a ligand of RAGE (Receptors for advanced glycation end products) to activate the NF-κB signaling pathway, thereby promoting the progression of CRC (39). Therefore, although several parasites have been proven to exert varying degrees of promotional effects on various types of tumor cells, their mechanisms of action are diverse. In our study, *S. japonicum* SEA promotes EMT and activates the MAPK and PI3K/AKT/mTOR signaling pathways in CRC cells, thereby promoting the malignant progression of CRC.

Autophagy has complex effects on CRC (40). On one hand, autophagy promotes tumor cell death and inhibits the development of CRC in early tumorigenesis. On the other hand, in the late stage of tumor progression, autophagy promotes tumor cell survival and enhances its resistance to drugs (40). We confirmed that *S. japonicum* SEA inhibits autophagy in CRC, and the autophagy activator RAPA can partially reverse the tumor-promoting effect of SEA on CRC. This indicates that SEA inhibits autophagy by activating mTOR, thereby promoting the development of CRC.

When studying the effect of SEA on autophagy, there were no changes when the cells were stimulated with SEA alone. We believe this result aligns with established autophagy regulatory biology. Under nutrient-rich conditions, active mTOR signaling suppresses autophagy, resulting in inherently low basal autophagic flux in CRC cells (41). The narrow dynamic range limits the detection of further inhibition of autophagy by SEA via western blotting. RAPA potently releases the brake on autophagy to significantly elevate basal autophagic flux (42), establishing a high-baseline state. In this context, the inhibitory effect of SEA acts on an amplified signal, and the resultant reduction is robust enough for reliable detection.

The composition of SEA is rather complex and contains various functional proteins. For instance, IPSE/α1 enters the cell nucleus and binds to DNA, while Omega-1 has ribonuclease activity (43). Intraperitoneal injection of *S. japonicum* SEA component SjE16.7 induces more and larger colon tumors in a mouse CRC model (39). Although our study demonstrated that the SEA mixture of *S. japonicum* promotes the growth of CRC both *in vitro* and *in vivo*, further research is needed to determine which specific component(s) in SEA are responsible for the tumor-promoting effect. While no reports have indicated that 3 mg/kg of SEA causes systemic toxicity in mice, our results showed reduced body weight in the SEA-treated mice, suggesting that SEA may have potential impacts on mouse physiology. We hypothesize that SEA promotes tumor growth by activating signaling pathways, which, in turn, leads to weight loss in mice. In addition, our *in vivo* experiments utilized an immunodeficient mouse xenograft model, which cannot recapitulate the complete host immune microenvironment or the chronic exposure to SEA that occurs during natural schistosome infection—this represents a limitation of the present study.

In conclusion, our study indicates that *S. japonicum* SEA induces the MAPK and PI3K/AKT/mTOR pathways and inhibits autophagy to promote the malignant progression of CRC. These findings provide a plausible mechanistic foundation for the long-observed clinical correlation between schistosomiasis japonica and both increased CRC incidence and poorer patient prognosis. Future research will explore whether *S. japonicum* infection leads to stronger activation of MAPK or PI3K-AKT pathways in tumor tissues of CRC patients. Correlating these molecular signatures with clinical outcomes (e.g., tumor stage, recurrence rate, survival data) is expected to establish SEA-associated signaling as a prognostic biomarker for schistosomiasis-associated CRC patients. Collectively, these results provide experimental evidence for elucidating the impact of *S. japonicum* infection on CRC and further deepen our understanding of the role of SEA in promoting tumor progression. Our study highlights the importance of schistosomiasis control as a potential strategy for CRC prevention in endemic regions.

## Data Availability

The original contributions presented in the study are included in the article; further inquiries can be directed to the corresponding authors.

## AFTER EPUB

*[This article was published on 2 March 2026 with an error in the Abstract. The Abstract was corrected in the current version, posted on 4 March 2026.]*
